# Aquaporin‐4 Orchestrates Muscle Cell Differentiation and Satellite Cell Preservation: A Novel Target in Age‐Related Muscle Decline

**DOI:** 10.1002/jcsm.70412

**Published:** 2026-10-27

**Authors:** María D. Girón, Manuel Manzano, Suzette L. Pereira, Antonio Reinoso, Chiara Burgio, Nora Montero‐Martin, Guillermo Jiménez‐Jiménez, Josep M. Argilés, Ricardo Rueda, José M. López‐Pedrosa, Rafael Salto

**Affiliations:** ^1^ Department of Biochemistry and Molecular Biology II, School of Pharmacy University of Granada Granada Spain; ^2^ Abbott Nutrition R&D Abbott Laboratories Granada Spain; ^3^ Abbott Nutrition R&D Abbott Laboratories Columbus Ohio USA; ^4^ Department de Bioquimica I Biologia Molecular, Facultat de Biologia Universitat de Barcelona Barcelona Spain

**Keywords:** AQP4, aquaporins, muscle proliferation and differentiation, sarcopenia, satellite cells, senescence, water transporters

## Abstract

**Background:**

Impaired muscle regeneration is a key contributor to age‐related muscle decline, yet the molecular mechanisms linking muscle water homeostasis to regenerative failure remain incompletely understood. Aquaporin‐4 (AQP4), a major skeletal muscle water channel, may have functions beyond water transport that are relevant to muscle maintenance and repair. We investigated whether altered AQP4 expression contributes to age‐related defects in skeletal muscle regeneration and whether AQP4 may represent a targetable pathway for muscle dysfunction associated with ageing.

**Methods:**

Skeletal muscle from young adult (25‐week; *n* = 9) and aged (85‐week; *n* = 8) rats was analysed for extracellular and intracellular water content by bioimpedance. AQP4, AQP1 and myogenic markers were measured by Western blot. Satellite cells isolated from skeletal muscle were assessed for proliferation, differentiation and expression of differentiation markers. To define the mechanistic role of AQP4 in myogenesis, CRISPR‐generated AQP4‐silenced L6 rat myoblasts were evaluated for proliferation, differentiation, senescence and Akt/PKB signalling. Rescue experiments were performed by restoring AQP4 expression in silenced cells. Data are reported as mean ± SEM.

**Results:**

Compared with young rats, aged rats showed an altered intracellular water‐to‐lean mass ratio (0.94 vs. 0.62, *p* < 0.05), a 33% reduction in AQP4 expression (*p* < 0.05) and reduced muscle strength (3.1 vs. 2.6, *p* < 0.05), with AQP4 levels correlating with muscle performance. Satellite cells from aged muscle exhibited 29% lower AQP4 expression and 39% lower Pax7 expression (both *p* < 0.05), together with reduced myogenic differentiation (30% decrease, *p* < 0.05). In L6 myoblasts, AQP4 silencing reduced proliferation (20% decrease, *p* < 0.05), impaired myotube differentiation (50% decrease, *p* < 0.05), increased senescence (30% increase, *p* < 0.05) and decreased Akt/PKB signalling by 42% (*p* < 0.05). AQP4 silencing was also associated with decreased dystrophin localization at the plasma membrane (50%, *p* < 0.05). Re‐expression of AQP4 reversed these abnormalities and restored the phenotype toward wild‐type levels (all *p* < 0.05).

**Conclusions:**

Reduced AQP4 expression is associated with impaired muscle regeneration and functional decline in aged skeletal muscle. Experimental AQP4 loss disrupts myogenic signalling, membrane organization and increases progression to senescence, whereas AQP4 restoration rescues these defects. These findings identify AQP4 as a biologically plausible therapeutic target for strategies aimed at preserving or restoring muscle health and regenerative capacity in ageing‐associated muscle dysfunction.

## 1 Introduction

Water is a vital component of human physiology, partitioned into extracellular (ECW) and intracellular compartments (ICW), with skeletal muscle exhibiting a particularly high‐water content. Ageing is associated with a progressive decline in total body water, especially ICW, which may reflect reductions in muscle cell mass and contribute to diminished muscle strength and function [1, 2]. Older adults are especially prone to dehydration, further exacerbating these changes. Recent evidence links ICW within lean mass (LM) with improved muscle strength, functional performance and reduced frailty [3]. Therefore, ICW/LM ratio has emerged as a promising biomarker of muscle quality and sarcopenia risk [1]. More recently, the appendicular ECW‐to‐ICW ratio has been independently associated with all‐cause mortality in middle‐aged and older adults, highlighting its clinical relevance in ageing populations.^1^

Water transport across the plasma membrane is regulated at least in part by aquaporins (AQPs) [4]. AQPs are small integral plasma membrane proteins responsible for passive water transport to counteract osmotic gradients in the cells [5]. Therefore, processes that involve water homeostasis, such as fat metabolism, cell proliferation, migration and adhesion, are regulated by them [6] and the modulation of AQPs activity is considered a possible target for the treatment of several diseases [7].

AQPs are widely distributed across mammalian tissues, including fluid‐transporting epithelia and endothelia, as well as cells not primarily involved in fluid exchange, such as erythrocytes, leukocytes, adipocytes and skeletal muscle. They are also expressed in astrocytes and glial cells within the central nervous system and sensory organs. AQPs are now understood to play diverse roles in fluid regulation, secretion, signal transduction, immune response, inflammation and tissue remodelling, reflecting their complex physiological significance [6].

AQP1 and AQP4 are the water channels identified to date in skeletal muscle, of which AQP4 is better studied [8, 9]. AQP4 is predominantly expressed in the sarcolemma of fast‐twitch muscle fibres and has been studied in the context of Duchenne muscular dystrophy [10] and other neuromuscular disorders [11]. In addition, some studies have related the AQP4 expression to exercise‐induced skeletal muscle adaptations [12]. AQP4 expression was also linked to muscle atrophy induced by rotator cuff injury [13]. However, not much is known about the role of AQP4 in ageing muscle and ageing‐related muscle dysfunction.

An important consequence of ageing is progressive loss of muscle mass, strength (termed Sarcopenia) along with an associated loss of functional capacity, metabolic dysfunction, reduced quality of life and ultimately increased mortality rate [14]. It is also well known that compared to young muscle, aged muscle has delayed/incomplete recovery from immobilization or muscle damage [15, 16]. Skeletal muscle contains designated stem cells (also called satellite cells) that proliferate only sporadically to replace myofibres damaged by daily activity and maintain the stem cell pool. Activation of satellite cells is essential for muscle regeneration since mature muscle cells (myofibrils) are terminally differentiated. Under normal conditions, in response to a stimulus (e.g., exercise, injury, remobilization), these satellite cells are activated, proliferate and differentiate to restore myofibrils [17, 18]. Researchers agree that the proliferative potential and repair capacity of these cells decline with ageing [17]. Differentiation of satellite cells into myofibres mostly depends on the Akt signalling pathway [17] that regulates the expression of differentiation markers. In ageing muscle, the process of myoblast differentiation appears to be disrupted and this is associated with reduced Myf5 expression [18].

The main objective of this study is to explore whether a downregulation of AQPs in skeletal muscle represents a causative factor in the development of muscle atrophy or merely a downstream consequence of pathological muscle degeneration. Therefore, we investigated the role of AQP4 in aged versus young rats and tested the impact of decreased AQP4 expression on the differentiation of myoblasts into myotubes in muscles and isolated satellite cells. Furthermore, the molecular bases of the processes involved in cell proliferation, differentiation and senescence, membrane integrity and protein associations were analysed using a rat muscle L6 cell line where the AQP4 expression was silenced.

## 2 Methods

### 2.1 Animal Studies

Male Wistar rats were used in this study. Animals were either 25 weeks old (young/adult) or 85 weeks old (aged). Two experimental protocols were conducted. In the first protocol, young/adult and aged rats were used to assess age‐related differences under basal conditions. In the second protocol, an additional group of 25‐week‐old (young/adult) rats received either vehicle or dexamethasone (100 g/kg body weight, intraperitoneal injection) once daily for 21 days to establish a model of acute muscle damage. All animals were fed the AIN‐93 M diet ad libitum for the experimental period. Throughout the study, animals were maintained under standardized environmental conditions, including a 12‐h light/dark cycle (lights on at 07:00), controlled ambient temperature (22°C ± 2°C), relative humidity (55% ± 10%) and an air renewal rate of 20 volumes per hour.

All experimental procedures (approval code 16.02.2024.029/27‐11‐2020‐137) were carried out according to the European Convention for the Protection of Vertebrate Animals used for Experimental and other Scientific Purposes (Directive 2010/63/EU) and the ethical guidelines for animal experimentation provided by the Spanish National Research Council (RD 53/1 February 2013).

### 2.2 Analysis of Grip Strength

A Bioseb GRIP TEST Version 3+ device was used to measure grip strength (Bioseb, Chaville, France). Both forepaws of the rat were placed on the front bar. Animals, held by the tail, grasped the bar and were gently pulled away in a smooth, steady motion until they released it. The strain gauge measured the force (N) needed to break the grip. Five readings were taken for each animal and, the highest force was recorded as that rat's grip strength score. All measurements were done blind to the experimental group.

### 2.3 Electrical Bioimpedance and Lean Body Mass Measurements

Total body water and intracellular fluid were measured by electrical bioimpedance as described [19] using an ImpediVET device (ImpediMed Inc., Pittsford, New York, USA). Body lean mass was measured through magnetic resonance imaging (MRI) using an Echo MRI Body Composition Analyser system (EchoMRI‐700, Echo Medical Systems, Houston, Texas, USA).

### 2.4 Isolation of Satellite Cells

Satellite cells were isolated from the adult and old rats' gastrocnemius muscle. The muscles were minced and digested for 2 h in Ham's F12 medium supplemented with 10% foetal calf serum (FCS) and 0.17% pronase at 37°C. The digested muscles were centrifuged first at 90*g* for 3 min and then the supernatant was centrifuged at 350*g* for 20 min to precipitate the satellite cells. Satellite cells were seeded in gelatin‐coated plates (0.5% in water). These cells grow in DMEM supplemented with 10% FCS and 10% horse serum (HS), penicillin and streptomycin. Subcultures enabled us to obtain an adequate number of cells for the various assays. For differentiation experiments, cells were incubated with 2% horse serum media for 5 days.

### 2.5 Culture of Skeletal Muscle Cells

These cell lines were provided by the cell culture facility of the University of Granada. The L6.C11 rat skeletal muscle myoblast line (ECACC No. 92102119) and the C2C12 murine skeletal muscle cells (ATCC CRL‐1772) grow in DMEM supplemented with 10% (v/v) FBS, 2 mmol/L glutamine plus 100 units/mL penicillin and 0.1 mg/mL streptomycin in an atmosphere of 5% CO_2_ and 95% air. It was maintained at subconfluent densities in the growth media. Cells were differentiated into myotubes by culturing them for 3 days in DMEM containing 2% FBS (v/v), changing the medium daily.

### 2.6 Inactivation of AQP4 by CRISPR‐Cas9

AQP4‐silenced cells were generated using a guide RNA (gRNA) for inducing a double‐strand break in the exon 2 of the rat AQP4 gene: 5′‐cggtggccacatcaacccagcgg‐3′, where the PAM sequence is underlined. The sequence was designed using https://chopchop.cbu.uib.no/ [20]. The gRNA without the PAM sequence was cloned into the CRISPR‐Cas9 plasmid PX459 (pSpCas9(BB)‐2A‐Puro, Addgene #62988) [21] to render plasmid PX459‐AQP4.

L6 myoblasts were transfected with the PX459‐AQP4 using Lipofectamine 2000. The transfected cells were grown for 2 days and then selected with puromycin for an additional 2 days. The surviving cells were clonally diluted into 96‐well plates to generate clonal populations from single cells.

Additionally, for the silencing of AQP4 expression in the murine C2C12 cell line, a gRNA that hybridises in the first exon of the mouse AQP4 gene (5′‐gtgggatccaccataaactgggg‐3′) was selected and a silencing plasmid based on PX459 was constructed as described above.

Western blotting was used to analyse isolated clones using an anti‐AQP4 antibody. Furthermore, from the wild‐type cells and positive clones, genomic DNA was isolated to PCR amplify the sequence flanking the indel introduced and the silencing was confirmed by Sanger sequencing [22].

### 2.7 Cloning and Eukaryotic Expression of Human AQP4

The coding sequence of the human AQP4 isoform M23 (Genebank #NM_001364286.1) was amplified by PCR using the oligonucleotides hAQP4F (5′‐agatctatggtggctttcaaaggggtctgg‐3′) and hAQP4R (5′‐ggtacctactgaagaacaatacctctccagatt‐3′) and retrotranscribed total RNA from human neuroblastoma SH‐SY5Y cells (ECACC No. 94030304) as a template. The sequences of these oligonucleotides include *Bgl*II and *Kpn*I restriction sites (underlined), respectively. The PCR product was cloned into the pJET 1.2 vector (Thermo Fisher Scientific, Madrid, Spain) and then digested with *Bgl*II‐*Kpn*I and cloned into the pEGFP‐N3 eukaryotic expression vector (Clontech Laboratories, Palo Alto, California). The sequence of the generated plasmids was confirmed by automatic sequencing with universal primers and was termed pEGFP‐hAQP4. Since the hAQP4 cloned upstream of the eGFP sequence lacks a stop sequence, the eGFP is expressed in the fusion protein at the cytosolic face of the plasma membrane. L6 cells not expressing AQP4 (L6 ΔAQP4) were transfected with pEGFP‐hAQP4 using Lipofectamine 2000.

### 2.8 Cell Proliferation Assay

A total of 1.6 × 10^5^ cells/well were seeded in 48‐well plates and grew for 72 h. Proliferation was evaluated using the 3‐(4,5‐dimethylthiazol‐2‐yl)‐2,5‐diphenyl‐2H‐tetrazolium bromide method. The results were calculated as the percentage of cell proliferation based on the 0‐h control cells. Additionally, cell proliferation was assayed using the CyQUANT Direct Cell Proliferation Assay Kit (Invitrogen, Paisley, UK), a nucleic acid stain‐based assay, following manufacturer instructions.

### 2.9 Determination of Protein Synthesis and Degradation

Muscle Protein synthesis was measured as described [23]. Data were computed as dpm/μg of proteins.

To measure protein degradation, L6 myoblasts were labelled with 1 μCi/mL of L‐[ring‐3, 5–3H]‐tyrosine (Tyr) as described [23].

### 2.10 Plasma Membrane Isolation

A plasma membrane‐enriched fraction from myotubes was prepared as described [24]. Caveolin expression was used as a marker for plasma membrane enrichment.

### 2.11 Protein Analysis

To study the expression and phosphorylation status of the proteins involved in metabolic and signalling events, L6 myoblasts or myotubes were processed as described previously [25].

Protein concentration was measured using the bicinchoninic acid method. Proteins (40 μg) were separated by SDS‐PAGE, transferred onto nitrocellulose membranes and immunoblotted with specific antibodies (Supporting Information Table S1). To assay the phosphorylation degree of key kinases, antibodies against phosphorylated proteins were used. To determine the expression of other proteins, the amount of GAPDH was used as a loading control. The immunoblots were developed using fluorescent secondary antibodies (Bio‐Rad, Madrid, Spain).

### 2.12 Plasma Membrane Water Flux

Cellular water content was assayed as described [26]. In brief, L6 or L6 ΔAQP4 cells were seeded and 24 h later, incubated with 5 μM Calcein AM (calcein acetoxymethyl ester) for 90 min at 37°C. Calcein is a fluorescent dye that is quenched in a water concentration‐dependent approach. Then, cells were washed and incubated with 0 or 400 mM Mannitol. Fluorescence (Excitation 485 nm, emission 520 nm) was measured every 5 min for 20 min in a Tecan Infinite 200Pro Plate Reader (Tecan Group Ltd., Männedorf, Switzerland). Then, mannitol was eliminated, cells were washed and fresh medium was added. Fluorescence was measured for an additional 20‐min period.

### 2.13 Osmotic Stress Assay

L6 or L6 ΔAQP4 cells were seeded on coverslips. 24 h later, they were incubated with 400 mM mannitol for 5 h. Cells were washed and fresh medium was added. Samples were taken at 0 h, post‐mannitol challenge and 24 and 48 h after changing the medium.

Coverslips were stained with Orange CellMask Plasma Membrane Stain (Thermo Fisher Scientific, Waltham, Massachusetts, United States) for 10 min. The cells were fixed with 2% paraformaldehyde and mounted for confocal microscopy.

### 2.14 Immunocytochemistry

To perform the immunocytochemistry procedure, cells were seeded on coverlips and 24 h later, were processed or differentiated for 3 days. The supernatant from each chamber was aspirated and the cells were rinsed twice with PBS. Then, the cells were fixed with 4% paraformaldehyde for 15 min and rinsed five times with PBS.

Next, the cells were permeabilized with 1% Triton‐X‐100 (diluted in PBS with 0.1% Tween‐20) for 10 min at room temperature with shaking. The cells were rinsed three times with PBS plus 0.1% Tween‐20. They were coated with 3% goat serum in 0.1% Tween‐20 PBS solution for 60 min at room temperature.

The solution from the wells was removed and 1/100 primary antibody (mouse anti‐AQP4 and/or rabbit anti‐Dystrophin; Pax 7 and anti‐Myf5, Data S1; rabbit anti‐Myf5 and anti‐Myf6, Figure S5) diluted in 0.5% goat serum, 1% BSA in 0.1% Tween‐20 PBS solution was added. They were incubated at 2°C–8°C overnight. The cells were rinsed 10 times in the PBS‐Tween and treated with a secondary antibody (Alexa Fluor 488 goat‐anti mouse and/or Alexa Fluor 594 goat‐anti rabbit) diluted in the same solution as the one used with the primary antibodies for 60 min at room temperature. The cells were rinsed five times with the D‐PBS‐Tween solution and stained with a Hoechst solution (0.5 μg/mL) for 2 min.

### 2.15 Cell Senescence β‐Galactosidase Staining

The protocol for detecting senescent cells involves staining with X‐gal (5‐bromo‐4‐chloro‐3‐indolyl‐β‐d‐galactopyranoside) at pH 6.0. The process includes fixing cells, washing and incubating them with the X‐gal staining solution overnight, at 37°C in the dark. The blue colour, detected by microscopy, indicates the presence of beta‐galactosidase activity as a marker of cellular senescence [27].

### 2.16 Confocal Microscopy

For confocal cell analysis, the coverslips were mounted on glass slides using ProLong Gold Antifade Reagent (Thermo Fisher Scientific, Waltham, Massachusetts, USA). Confocal microscopy was performed on a Leica TCS‐SP5 II multiphoton confocal microscope (Central Facilities, University of Granada, Spain), using a sequential acquisition mode to separately collect images in individual channels for colour analysis. A pinhole of 1 Airy unit was utilized. Images were acquired at a resolution of 1024 Å. Series were captured in the xyz mode. Data were processed using the Leica Application Suite software package.

### 2.17 Statistical Analysis

Results are expressed as mean ± SEM. Statistical analysis was performed using one‐ or two‐way ANOVA, followed by Tukey's test as appropriate. When comparing two variables, a Student's *t*‐test was used. *p* < 0.05 was considered statistically significant.

## 3 Results

### 3.1 Expression of AQP4 Correlates With Muscle Functionality in an In Vivo Ageing Model

Ageing leads to a reduction in skeletal muscle mass and function, contributing to sarcopenia and is accompanied by impaired cellular water transport [1, 3, 28, 29]. Using an ageing animal model, we investigated the role of AQPs in muscle degeneration beyond their canonical function in water homeostasis. Young adult and old rats were used to compare grip strength, followed by analysis of their gastrocnemius muscle to determine differentiation markers, proteins related to muscle protein turnover and performance, water content and expression of AQPs.

Grip strength and creatine kinase (CK), a marker of late myoblast differentiation, were higher in young adult rats than in older rats (Figure 1A). Additionally, the expression of myoblast differentiation markers such as Pax7, Myf5 and Myf6 (Figure 1B) indicated that the muscles from older rats had lower levels of differentiated myoblasts, indicating less regenerative potential than younger rats. Moreover, when analysing muscle protein turnover signalling markers, a decrease in mTOR phosphorylation and an increase in protein degradation (reflected by the levels of Atrogin and MurF1) were detected in the older rats compared to young adult rats. In parallel, the intracellular water content and total body water (Figure 1D), measured by bioimpedance, were significantly reduced in the older rats' muscles compared to those of young adult rats. Furthermore, the ICW/LM ratio, considered a biomarker of muscle quality and sarcopenia risk, was 35% reduced in the older rats. In parallel, the older rats' muscles had a significantly lower amount of AQP4 (Figure 1E) compared to adult rats (33%), but the levels of AQP1 were similar between the two groups.

**FIGURE 1:**
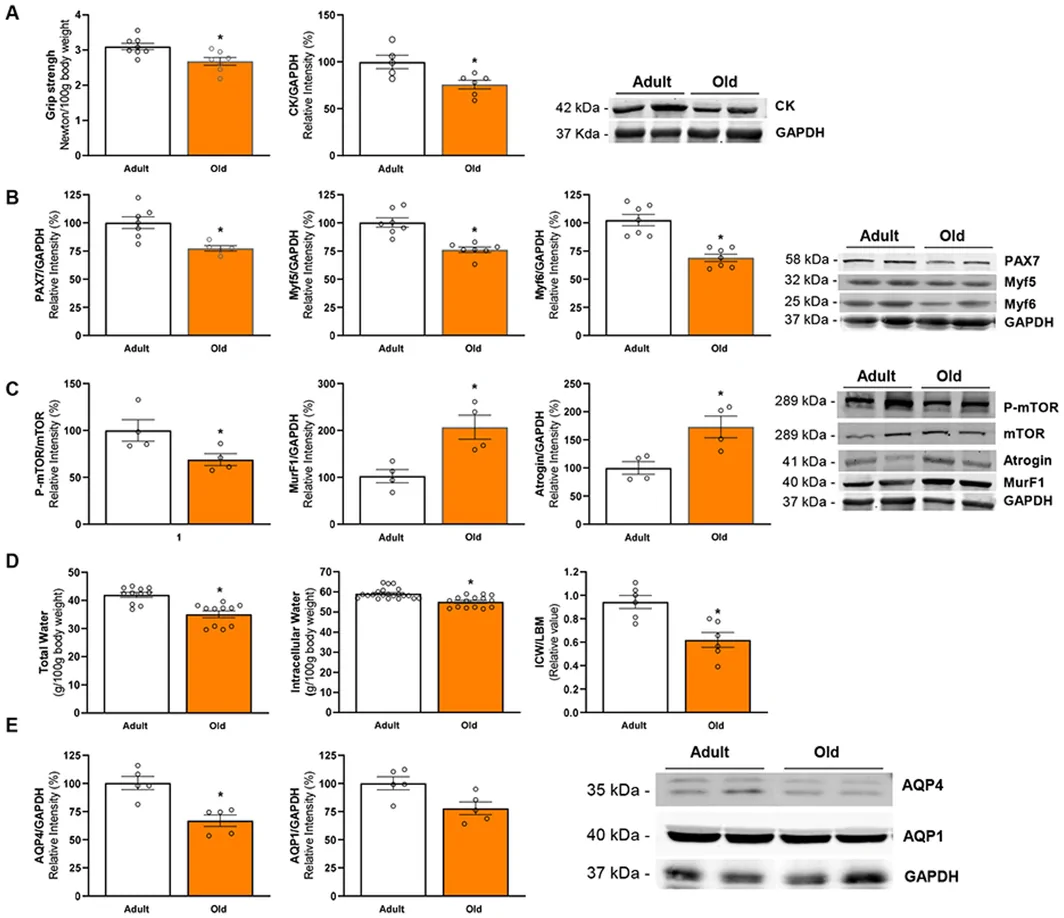
Ageing muscle is characterized by lower strength, lower myoblast differentiation, increased protein degradation associated with lower water content and AQPs expression. (A) Measurement of Grip strength in young adult (*n* = 9) and old (*n* = 8) rats and expression of Creatine Kinase (CK) (adults, *n* = 5; olds, *n* = 6). (B) Expression of myoblast differentiation markers (Pax7, Myf5 and Myf6) (*n* = 6). (C) Protein turnover: synthesis (activation of mTOR) and degradation (expression of Atrogin and MurF1) (*n* = 4). (D) Muscle total, intracellular water content (adults (*n* = 23); olds (*n* = 21)) and ICW/LBM relative values. (E) Expression of AQP4 and AQP1 transporters (*n* = 6). Gastrocnemius muscle lysates from adult and old rats were used to measure the expression of different proteins. mTOR (for P‐mTOR) or GAPDH was used as a loading control. Data are mean ± SEM. *p < 0.05 vs. adult muscles.

Analysis of AQP expression in slow‐twitch (soleus) and fast‐twitch (EDL) muscles showed that ageing reduced AQP4 expression in both muscles, with a greater reduction in the fast‐twitch EDL than in the slow‐twitch soleus (Figure S1).

### 3.2 Satellite Cells From Old Rats' Muscles Show Decreased Proliferation Rates and an Unproductive Differentiation Pathway

Since the older rats' muscles showed decreased differentiation marker levels, satellite cells were isolated from the gastrocnemius muscle and the satellite cells capability to proliferate and differentiate and the expression of AQPs, were measured (Figure 2). AQP4 levels were significantly lower in the satellite cells from older muscles compared to those from adult muscles. In contrast, a significant increase in AQP1 was detected in these cells (Figure 2A). When the capability to proliferate was assessed, the older satellite cells showed decreased proliferation (Figure 2B) along with a lower expression of differentiation markers (Figure 2C). The co‐expression of differentiation markers in the satellite cells was evaluated by immunocytochemistry (Figure 2E) to identify individual satellite cells expressing two differentiation markers at the same time. The number of PAX7/Myf5^High^ as well as PAX7/Myf5^Low^ cells was lower in old satellite cells.

**FIGURE 2:**
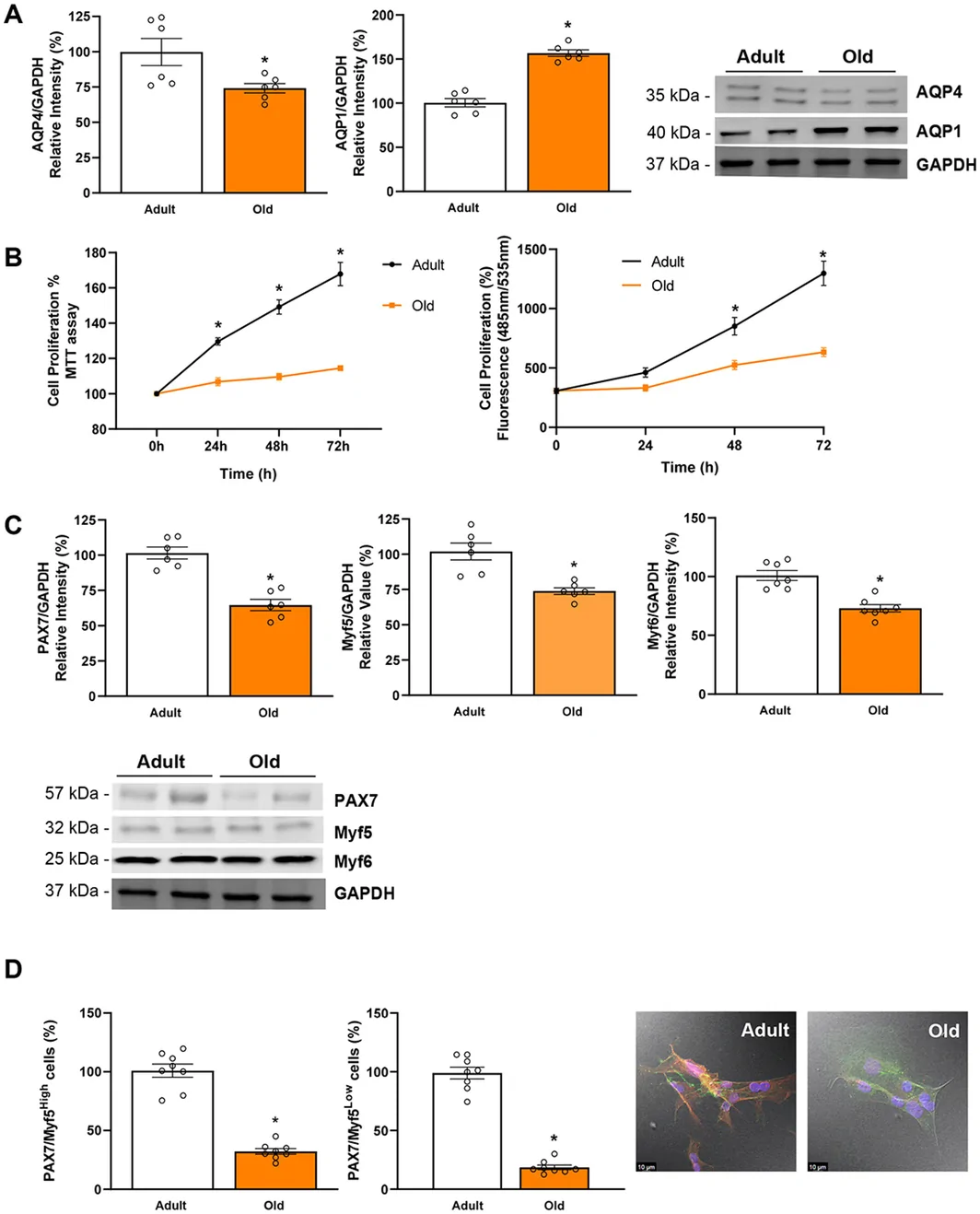
AQPs expression, proliferation and differentiation in satellite cells isolated from adults and old rats. (A) AQP4 and AQP1 expression levels. (B) Proliferation rate measured by a MTT assay and a CyQUANT Direct Cell Proliferation Assay (C) Differentiation markers levels. (D) Percentage of PAX7/Myf5High and PAX7/Myf5Low cells (normalized to 100% satellite cells from adult animals). Representative images of these cells are shown. Data are mean ± SEM. *N* = 6–8. *p < 0.05 vs. adult muscles.

Satellite cells isolated from adult and aged rats were differentiated in medium containing 2% horse serum and PAX7 and creatine kinase (CK) expression was assessed (Figure S2). Differentiation reduced PAX7 expression in both groups; however, CK induction, consistent with myogenic differentiation, was significantly greater in satellite cells from adult than from aged rats.

To further examine the association between AQP4 and myogenic regulation, we used a dexamethasone‐induced model of acute muscle damage. In dexamethasone‐treated animals, reduced AQP4 expression was accompanied by lower expression of PAX7, Myf5 and Myf6 in skeletal muscle (Figure S3).

### 3.3 Development of an AQP4 CRISPR Cell Model to Understand Its Role in Muscle

Considering the observed reduction in AQP4 expression concomitant with ageing‐associated muscle dysfunction, we decided to create a cellular model to investigate the relevance of AQP4 levels in sarcopenia. To achieve this, the rat muscle L6 myoblast cell line, a well‐characterized cell line that retains the capacity to differentiate into myotubes from myoblasts, was selected. In this cell line, the differentiation process induces the expression of AQP4 (Figure 3A), which is under the control of the Akt, ERK and mTOR signalling pathways as measured in the presence of specific inhibitors of each pathway (Figure 3C).

**FIGURE 3:**
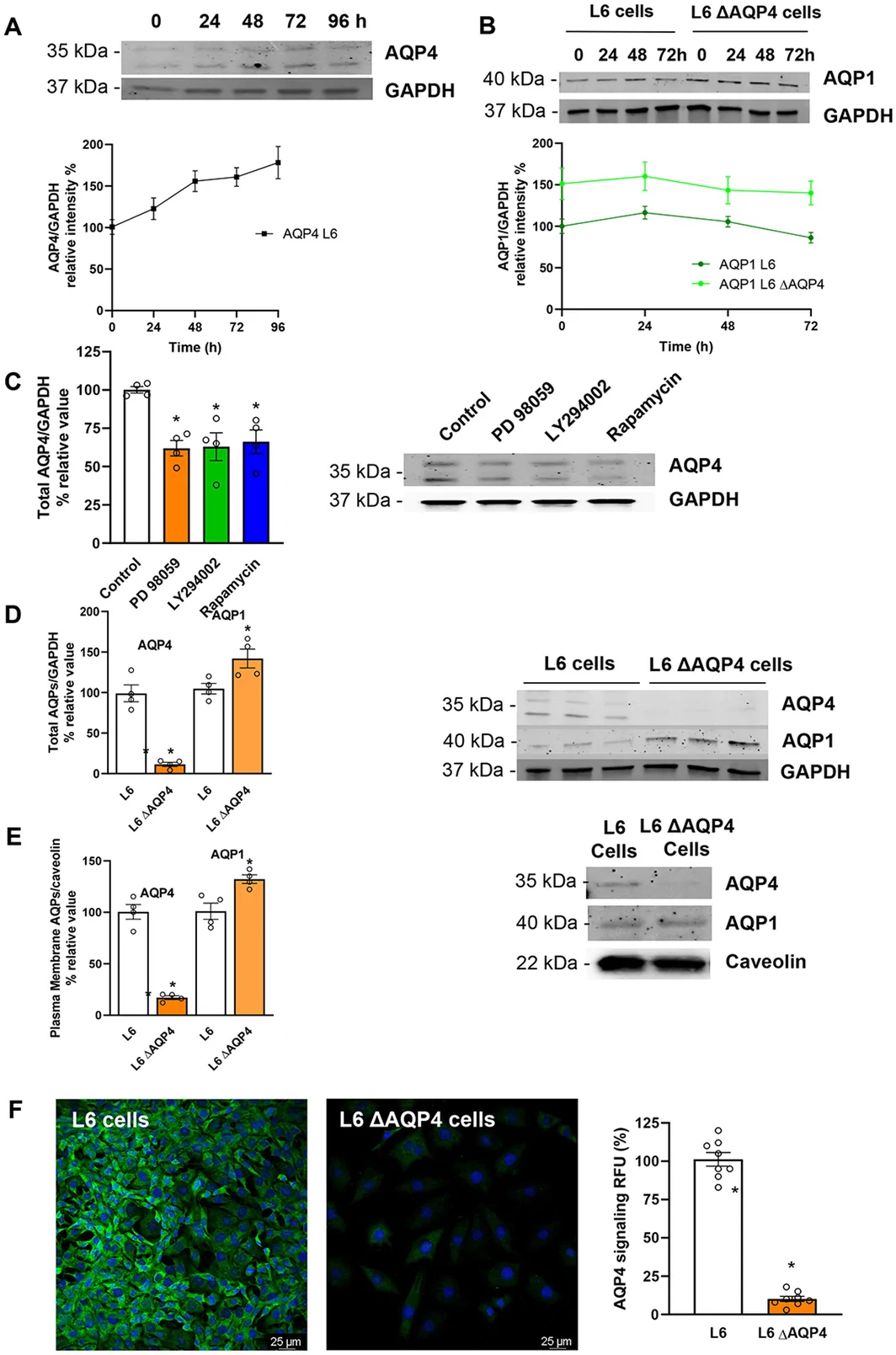
AQP4 silencing in the rat muscle L6 cell line. AQP4 (A) and AQP1 (B) expression levels during myoblast to myotube differentiation. (C) Effect of signalling pathway inhibitors on the expression of AQP4 in differentiated L6 myotubes. PD98059, LY294002, or rapamycin, each at 10 μM, were used as inhibitors of ERK1/2, Akt, or mTOR activation, respectively. Total AQPs expression (D) and plasma membrane AQPs expression (E) in normal (L6) and silenced (L6 ΔAQP4) cells. Data are mean ± SEM. *N* = 4–5. *p < 0.05 vs. control. (F) AQP4 immunocytochemistry of L6 and L6 ΔAQP4 cells. Cells were stained with an anti‐AQP4 antibody (green) and the nucleus (blue) was stained with Hoechst. Relative AQP4 staining intensity has been quantified. Data are mean ± SEM. *N* = 8. *p < 0.05 vs. adult muscles.

To examine the role of AQP4 in myoblast differentiation and functionality, an L6 isogenic cell line (L6 ΔAQP4) was generated, in which CRISPR has obliterated the expression of AQP4. The blockade of AQP4 expression was confirmed by Western blot and immunocytochemistry (Figure 3D,E,F). Interestingly, AQP4 depletion increased AQP1 expression (Figure 3B,D), as observed in satellite cells from aged muscle (Figure 2A).

### 3.4 AQP4 Is Needed for the Differentiation of Myoblasts Into Myotubes

Using the L6 ΔAQP4 cell line, we evaluated the influence of AQP4 silencing on cell proliferation and senescence. As shown in Figure 4, the lack of AQP4 translates into a lower proliferation rate than wild‐type L6 cells when grown in non‐differentiation media (Figure 4A). Similar cell proliferation results were obtained when analysing the silencing of AQP4 in C2C12 cells (Figure S5). Furthermore, the L6 ΔAQP4 cells showed a higher senescence rate, measured by the β‐galactosidase assay (Figure 4B) and the presence of cells with a morphology compatible with senescence (zombie cells) [30]. The higher senescence detected in the L6 ΔAQP4 cells is associated with a decrease in the expression of p16 and an increase in p21 levels, both markers of cell cycle arrest (Figure 4C).

**FIGURE 4:**
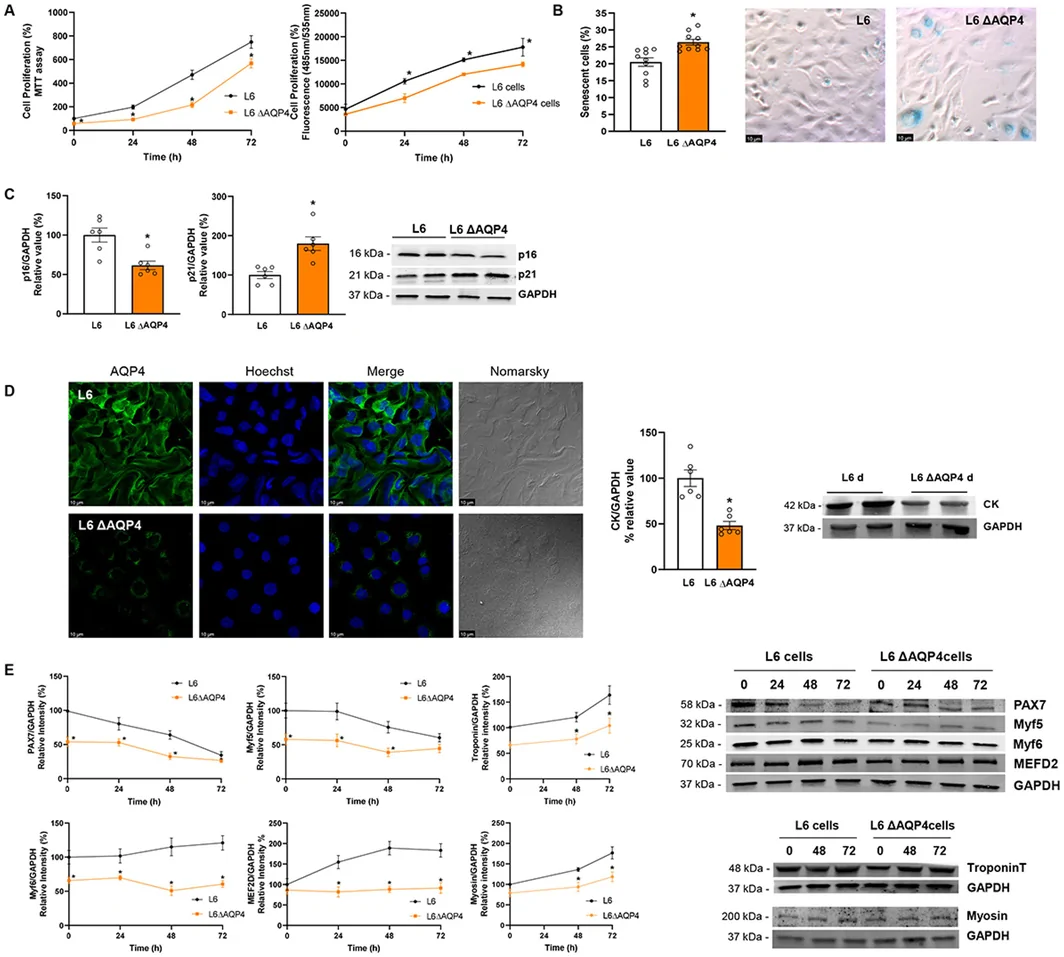
Effects of AQP4 silencing in the L6 cell line on cell proliferation, senescence and cell differentiation. (A) Proliferation rate (*n* = 8). (B) Percentage of positive β‐galactosidase cells and representative images (*n* = 10). (C) Expression levels of p16(CDKN2A) and p21(CDKN1A) as senescence markers (*n* = 6). (D) Confocal images of L6 and L6 ΔAQP4 cells upon differentiation. Cells were stained with an anti‐AQP4 antibody (green) and the nucleus (blue) was stained with Hoechst. CK levels have been measured as a marker of cell differentiation. (E) Expression levels of differentiation markers along the differentiation process (*n* = 4). Data are mean ± SEM. *p < 0.05 vs. L6 cells.

When both cell types are cultured in differentiation media, deficient differentiation of L6 ΔAQP4 cells is observed (Figure 4D). This result is corroborated when differentiation markers are analysed throughout the differentiation process. L6 cells show PAX7, Myf5, Myf6, MEF2D, Troponin T and Myosin profiles consistent with differentiation from myoblasts to myotubes. In contrast, no changes in these differentiation markers are observed over time in L6 ΔAQP4 cells, confirming a reduction in differentiation capacity (Figure 4E).

The effects of AQP4 silencing on C2C12 differentiation were analysed by immunocytochemistry. Myf5 and Myf6 expression was reduced in AQP4‐silenced C2C12 cells by 35% and 30%, respectively, compared to wild‐type C2C12 cells (Figure S5).

Since the differentiation of myoblasts to myotubes is driven by specific signalling pathways such as Akt or ERK pathways, we have analysed the activation of these pathways in both cell types. To promote differentiation, L6 cells show enhanced Akt and ERK signalling pathways, whereas in L6 ΔAQP4 cells, Akt and ERK activation is suppressed, as indicated by reduced AKT and ERK phosphorylation and GLUT4 expression (Figure 5A,B). In addition, the blockade of the Akt pathway in the L6 ΔAQP4 cells translates into decreased activation of mTOR and protein synthesis (Figure 5A,B). Also, increased protein degradation and an increase in degradation markers such as Atrogin and MurF1 are detected in these cells, leading to negative protein turnover (Figure 5B). When the signalling pathways are assayed in the differentiated L6 and L6 ΔAQP4 cells (Figure S4), similar results are obtained.

**FIGURE 5:**
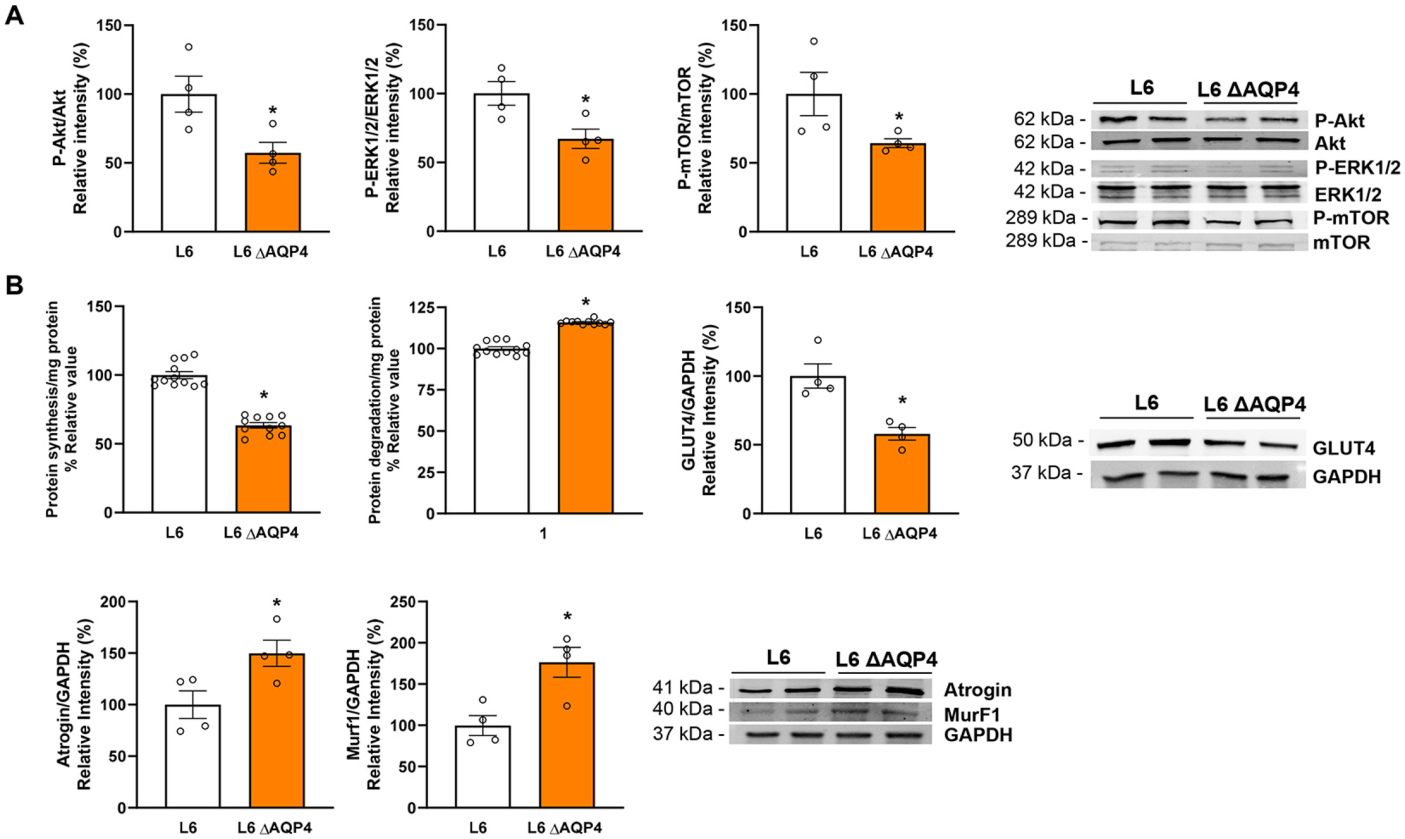
Effects of AQP4 silencing in the rat muscle L6 cell line on cell signalling, protein synthesis and protein degradation in undifferentiated myoblasts. (A) Akt, ERK and mTOR activation (*n* = 4). (*n* = 4). (B) Net protein synthesis (L6, *n* = 12; L6 ΔAQP4, *n* = 11), protein degradation (L6, *n* = 12; L6 ΔAQP4, *n* = 11), GLUT4, Atrogin and MurF1 expression levels (*n* = 4). Data are mean ± SEM. *p < 0.05 vs. L6 cells.

### 3.5 AQP4 Is Needed for Resistance to Osmotic Stress and the Preservation of the Plasma Membrane Structure and Integrity in L6 Cells

In the silenced L6 ΔAQP4 cells, there is an increase in the AQP1 expression levels (Figure 3B). Therefore, we conducted experiments to determine whether this increased AQP1 expression is enough to maintain cell viability and plasma membrane integrity under osmotic stress. We analysed intracellular water content and cell survival in response to mannitol‐induced osmotic stress. Figure 6A shows that although water content under non‐stress conditions is comparable between L6 and L6 ΔAQP4 cells, the latter exhibit reduced intracellular water levels under osmotic stress and an incomplete recovery once the stressor is removed. Additionally, L6 ΔAQP4 cells display consistently lower survival rates across all stressor concentrations (Figure 6B). When the effects of mannitol‐induced osmotic stress were analysed in differentiated cells, similar results were obtained (Figure 6C).

**FIGURE 6:**
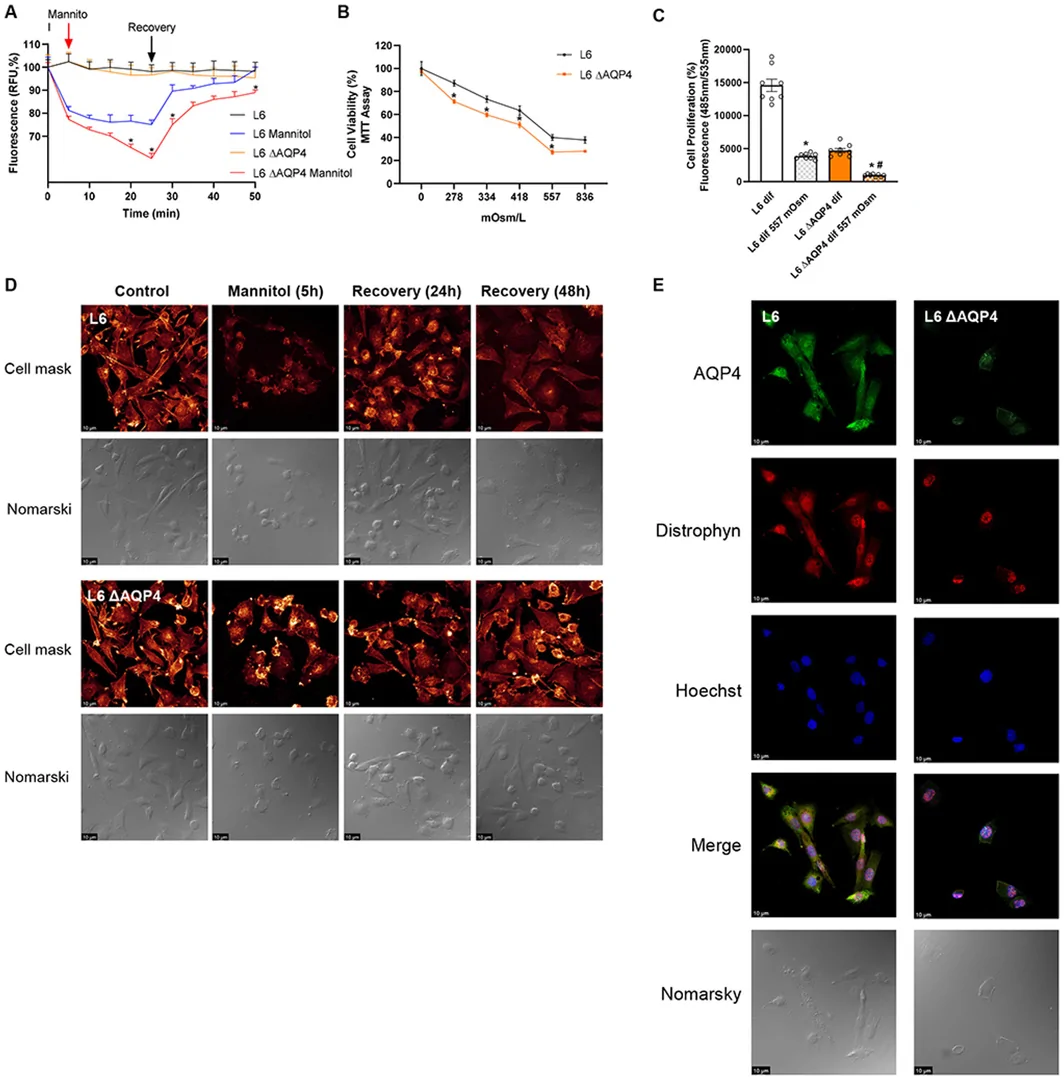
AQP4 role in resistance to osmotic stress, preservation of plasma membrane integrity and dystrophin subcellular distribution. (A) Calcein‐incubated L6 or L6 ΔAQP4 cells were treated with 0 mM or 400 mM mannitol and then recovered in fresh medium. Fluorescence was measured during the experiment. (*n* = 4) (B) Cell viability of L6 and L6ΔAQP4 cells incubated for 24 h with increasing concentrations of Mannitol in DMEM media (miOsm/L) (*n* = 8). (C) Cell viability of differentiated L6 and L6ΔAQP4 cells incubated for 24 h with Mannitol 557 miOsm/L in DMEM media (*n* = 8). (D) Images of L6 or L6 ΔAQP4 cells incubated with 400 mM mannitol for 5 h and recovered for 48 h with fresh medium. Cells were stained with Orange CellMask. (E) Subcellular distribution of AQP4 and dystrophin in L6 and L6 ΔAQP4. Subcellular distribution has been analysed by immunocytochemistry with antibodies anti‐AQP4 (green) and anti‐Dystrophin (red). The nucleus (Blue) was stained with Hoechst. Data are mean ± SEM. *p < 0.05 vs. L6 mannitol‐treated cells.

The decreased survival rate and restoration of intracellular water content in the L6 ΔAQP4 cells were directly associated with altered cell morphology and damaged plasma membrane integrity, which was detected when the cells were incubated with Cellmask Orange, a well‐known plasma membrane integrity marker (Figure 6D). The damage persists in the L6 ΔAQP4 cells even after removal of the osmotic stressor compared to L6 cells. Similar results were obtained when the propidium iodide uptake (a non‐permeant compound) was measured by confocal microscopy (Figure S6).

Muscle membrane integrity is based on the association of key proteins that maintain the cellular structure. One of these proteins is dystrophin, and therefore, we studied the subcellular distribution of AQP4 and dystrophin in L6 and L6 ΔAQP4 cells. Confocal analysis of the AQP4 and dystrophin distribution in these cells (Figure 6E) indicates that dystrophins have a diffuse localization in the L6 cells expressing AQP4, including the plasma membrane, cytosol and nucleus. On the contrary, in the L6 ΔAQP4 cells, dystrophin has only a nuclear distribution.

To further support our results, we have generated a eukaryotic expression vector in which the GFP protein is fused to the carboxy end of the human AQP4. The fusion protein has been described to retain its biological activity [5]. When the L6 ΔAQP4 cells were transiently transfected (Figure 7), the transfected cells could be detected by the fusion protein‐associated GFP fluorescence. In non‐differentiation media, the transfected L6 ΔAQP4 cells exhibit an increase in Pax7 expression compared to the non‐transfected cells (Figure 7A), consistent with the data obtained from young adult muscle satellite cells. In addition, L6 ΔAQP4 cells transfected with hAQP4‐GFP and stained with the plasma membrane Cell Mask display a well‐defined plasma membrane, like that observed in the wild‐type L6 cells (Figure 7B). Furthermore, in the transfected cells, dystrophin recovered its subcellular distribution, including within the plasma membrane, whereas in the non‐transfected cells, it was localized predominantly to the nuclear region (Figure 7C). Finally, when the cells were grown in differentiation media, only in the transfected green cells, cell fusion was detected, associated with the differentiation to myotubes (Figure 7D).

**FIGURE 7:**
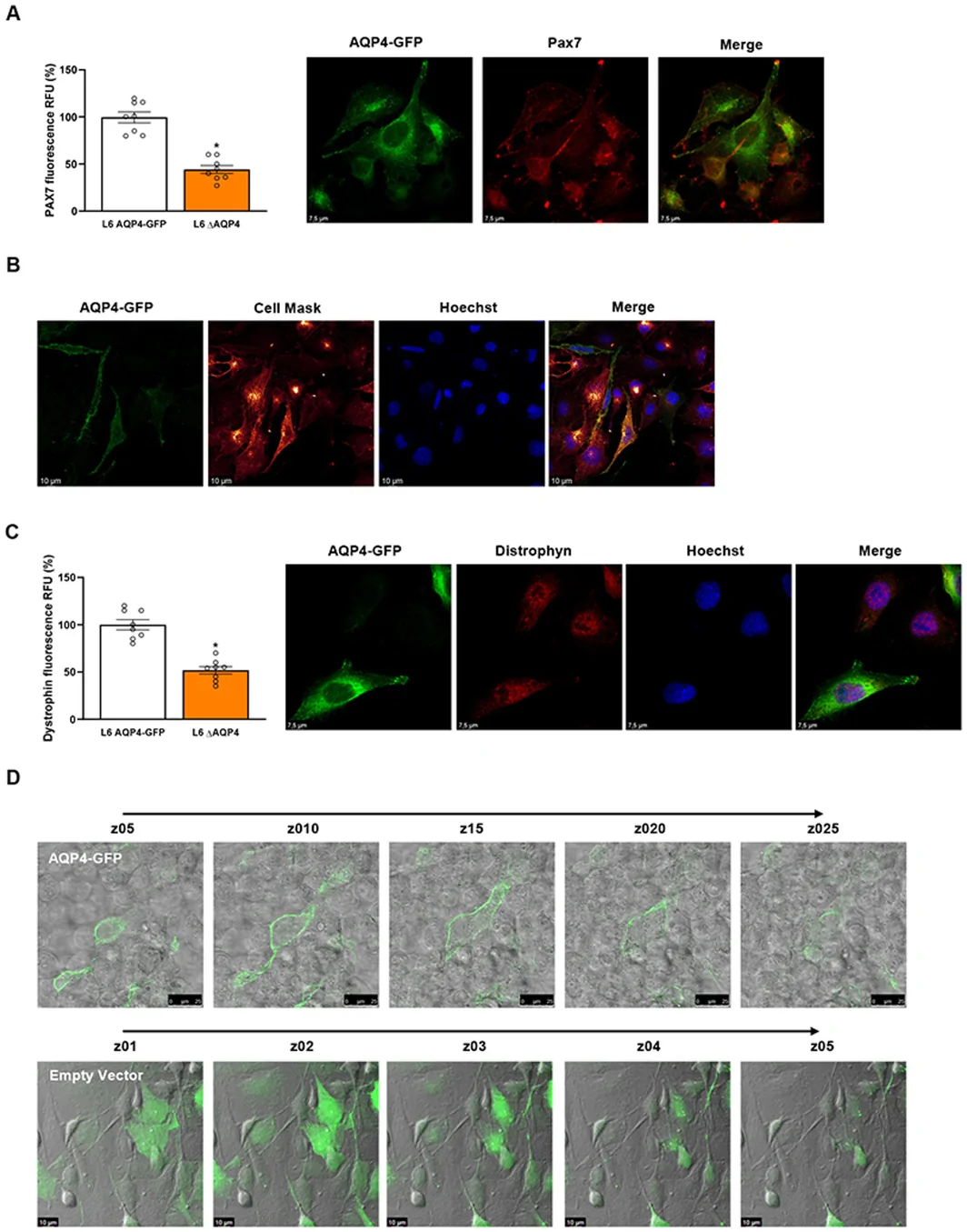
Phenotype recovery in L6 ΔAQP4 cells transiently transfected with a vector encoding for hAQP4‐GFP fusion protein. In the transient transfection, AQP4‐GFP has been detected by the green fluorescence of GFP. (A) Subcellular distribution of Pax7 (red) has been analysed by immunocytochemistry with anti‐Pax7 antibodies. The intensity of the PAX7 labelling in AQP4‐GFP and non‐transfected cells is shown. (B) Images of L6 ΔAQP4 cells transfected with AQP4‐GFP (green) and stained with Orange CellMask. The nucleus (blue) was stained with Hoechst. (C) Dystrophin subcellular distribution has been analysed by immunocytochemistry with antibodies anti‐AQP4 (green) and anti‐dystrophin (red). The nucleus (blue) was stained with Hoechst. The intensity of the Dystrophin labelling in AQP4‐GFP and non‐transfected cells is shown. (D) Series of images (z05–z025) of L6 ΔAQP4 cells transfected with AQP4‐GFP or the empty vector pEGPF‐N3 (green) and differentiated for 5 days in serum‐deprived medium.

## 4 Discussion

Ageing is associated with a decrease in total body water and, dehydration could be a determining factor in muscle performance. AQPs are integral membrane proteins traditionally recognized for facilitating passive water transport across biological membranes. However, emerging evidence indicates that their physiological roles are more complex [6]. Among these, AQP4 is the predominant isoform expressed in skeletal muscle, with a specific enrichment in the sarcolemma of fast‐twitch fibres [9]. AQP4 is thought to contribute to the regulation of myocyte water homeostasis and cell volume, particularly under conditions of osmotic or metabolic stress. Notably, AQP4 protein levels are significantly reduced in skeletal muscle following denervation, as well as in pathological conditions such as Duchenne muscular dystrophy and mechanical muscle injuries [9, 31, 32]. These observations support the hypothesis that AQP4 downregulation is associated with pronounced muscle atrophy, suggesting a potential mechanistic link between AQP4 expression and the maintenance of muscle mass and function. However, whether AQPs loss is a driving factor in the pathogenesis of muscle atrophy or a secondary consequence of muscle waste remains unresolved.

In this study, we employed a rat model of skeletal muscle ageing alongside cultured L6 rat skeletal muscle myoblasts to investigate the contribution of AQP4 in muscle regeneration and satellite cell function. This dual approach provides complementary insights into the molecular pathways and regulatory processes underlying AQP4‐associated muscle degeneration. The muscles of the ageing rats displayed features characteristic of human ageing muscle, including decreased muscle strength and markers of function (CK), decreased muscle protein synthesis (deduced from a reduced mTOR activation) and increased protein degradation (increased expression of Atrogin and MurF1 proteins) [33, 34, 35, 36]. Correspondingly, the aged rat muscle displayed decreased expression of AQP4 without significant changes in AQP1. As expected, the reduced expression of AQP4 paralleled observed decreases in muscle total and intracellular water content, given the well‐established role of AQPs in water transport. Surprisingly, the levels of AQP1 did not change significantly with age, possibly indicating that AQP4 plays a predominant role in water homeostasis. Although the decrease in AQP4 expression in these muscles has not been directly attributed to a decrease in satellite cells or also occurs in the sarcolemma of myofibres, in general our in vivo results support the role of AQP4 in muscle functionality.

Our data also point to the role of AQP4 in muscle regenerative capacity. When several differentiation markers were assayed, they were significantly reduced in the older rats´ muscles compared to young muscle, suggesting dysfunction in the process of forming new myofibres needed for regeneration. Using muscle satellite cells isolated from adult and old rats, similar results were obtained. First, a decreased proliferation rate was observed in satellite cells from old muscles. The lower proliferation was concomitant with a reduced AQP4 expression and a moderate increase in AQP1 levels. We analysed by immunocytochemistry the individual expression of PAX7 and Myf5 in each satellite cell and found a PAX7 subcellular distribution consistent with other studies that locates Pax7 in the nucleus but also in the cytosol or plasma membrane during myogenesis [37]. As shown in Figure 2D, immunocytochemical analysis identified two satellite cell subpopulations: one co‐expressing PAX7 and high levels of Myf5 (PAX7+/Myf5HIGH) and another expressing PAX7 with low levels of Myf5 (PAX7+/Myf5LOW). Both subpopulations were reduced in aged rats. The PAX7+/Myf5LOW population has been described as a quiescent reserve pool capable of proliferating and replenishing the satellite cell compartment, whereas PAX7+/Myf5HIGH cells represent a more committed myogenic population with the capacity to differentiate and contribute to muscle repair. The reduction in both populations with ageing suggests that older muscle has diminished regenerative and reparative capacity.

Furthermore, when the expression of a late differentiation marker such as Myf6 [38] was measured, a decreased level was detected in the older satellite cells. Satellite cells are responsible for skeletal muscle renewal. At rest, they enter a reversible state of quiescence without division. In response to a stimulus (e.g., injury/exercise), satellite cells can be activated and re‐enter the cell cycle, creating new cells to repair and create nascent muscle fibres, while preserving a small population that can return to quiescence for future regenerative demands. However, in ageing muscle, satellite cells that experience prolonged quiescence undergo programmed cellular senescence, an irreversible state of non‐division that impairs the regenerative capacity of the muscle [39].

Our results highlight a significant association between AQP4 and the regulation of muscle cell proliferation, differentiation, regeneration and protein turnover, underscoring its potential role as a critical modulator of skeletal muscle homeostasis and structural remodelling.

To further elucidate the importance of AQP4 in the regulation of muscle proliferation and differentiation, we have developed a cellular model using L6 rat muscle myoblasts. This cell line has been extensively used as a model to study muscle differentiation and functionality [40, 41]. Interestingly, upon differentiation to myotubes, there is an increase in AQP4 expression without significant changes in AQP1 amount over time. Furthermore, in the L6 cell line, the induction of AQP4 expression upon differentiation was dependent on the Akt and mTOR signalling pathways, two of the more relevant pathways able to drive the muscle differentiation process. Although direct evidence linking AQP4 expression to Akt/mTOR signalling in L6 myoblasts remains limited, our findings are consistent with the well‐established role of these pathways in regulating skeletal muscle differentiation. Given that AQP4 expression increases during myogenic progression and that Akt/mTOR signalling is a key driver of this process, it is plausible that AQP4 may be modulated downstream of these pathways. To validate this hypothesis, additional experiments were conducted by silencing AQP4 expression in L6 cells.

We silenced AQP4 expression in L6 cells using a well‐established CRISPR‐Cas9 technique [21]. In response to AQP4 silencing in the L6 ΔAQP4 cells, there is a moderate increase in AQP1 basal expression, which is not significantly modified during the differentiation process, similar to what was observed in the satellite cells from older muscles. Using this L6 ΔAQP4 isogenic cell line, we first analysed the relevance of AQP4 in cell proliferation. Replicating the behaviour detected by the old satellite cells, the L6ΔAQP4 cell line showed a lower proliferation rate in non‐differentiation media.

Ageing is classically related to enhanced senescence [42]. In skeletal muscle, senescence serves as a protective mechanism that limits the proliferation of damaged or dysfunctional cells. However, excessive accumulation of senescent satellite cells, “zombie cells,” can impair regenerative capacity by blocking cell proliferation, inhibiting myogenic differentiation and disrupting tissue repair. This dysfunctional state contributes to the ageing muscle phenotype, characterized by progressive loss of muscle mass and decline in contractile function [30].

In muscle, two main pathways are involved in senescence: the p16‐pRB and the p53‐p21 pathways. In our study, we assayed key elements in both pathways. While there is a decrease in p16 expression in silenced cells, there is a significant increase in p21 levels, suggesting that senescence is predominantly mediated through the p53‐p21 pathway. This shift may underline the reduced proliferative capacity observed in these cells. Interestingly, an enhancement of the p21 pathway has been described recently in animal and human models of muscle ageing [43, 44], supporting the idea that a decrease in AQP4 expression in ageing could be responsible for the molecular events described.

In L6 cells, the induction of AQP4 expression during the process of differentiation is dependent on the Akt and mTOR signalling pathways. Therefore, we analysed the activation of the Akt pathway and Akt‐dependent GLUT4 transporter expression, mTOR activation and net protein synthesis in L6ΔAQP4 cells. Our results indicated that L6ΔAQP4 cells lost the capacity to stimulate both signalling pathways, which consequently led to a decrease in glucose uptake and protein synthesis. In addition, probably due to the decrease in Akt signalling, an increase in protein degradation and degradation markers was observed in the L6ΔAQP4 cells. Altogether, the loss of AQP4 caused the development of a sarcopenic muscle phenotype, a hallmark of muscle ageing.

Interestingly, our results show that AQP4 can set a bidirectional regulatory circuit in skeletal muscle cells. On one hand, in L6 cells, AQP4 expression is modulated by the Akt and mTOR signalling pathways. On the other hand, our data in L6 ΔAQP4 indicate that AQP4 itself may influence the activation of these pathways. This reciprocal interaction could serve as an amplification system, whereby even small changes in AQP4 levels significantly impact muscle cell signalling dynamics, thereby affecting proliferation, differentiation and overall cellular functionality.

We also investigated the role of AQP4 in membrane integrity and water transport to further elucidate the importance of AQP4 in muscle health. Our studies revealed that under isotonic conditions, intracellular water levels are similar in both wild‐type and knockout cell lines; however, when cells are faced with a hyperosmotic stress, the ability of L6 ΔAQP4 cells to retain intracellular water decreased and recovery after stress was not complete. This situation mimics that detected in the ageing muscles, where repetitive dehydration events lead to a loss of muscle mass associated with damage‐induced senescence [45, 46, 47]. Both decreases in cell proliferation and loss of membrane integrity were observed under osmotic stress, which are not restored after removal of the stress. This points to a critical role of AQP4 in maintaining muscle integrity and muscle resilience to stress situations.

It is described that AQP4 and dystrophin are closely linked at the plasma membrane in muscle [10]. In fact, in Duchenne muscular dystrophy, it has been described that the lack of dystrophin and α1‐syntrophin is associated with a decreased expression of AQP4 at the plasma membrane that alters osmotic permeability [31]. In addition, in myoblasts lacking dystrophin, there is an impairment of the PI3K/Akt/mTOR signalling pathways and a higher activation of autophagy‐related genes, leading to an impaired differentiation programme [48].

Using immunocytochemistry, we analysed the subcellular distribution of AQP4 and dystrophin in the L6 and L6 ΔAQP4 cells. In L6 cells, dystrophin is primarily localized at the plasma membrane and diffuses in the cytosol; this location is compatible with the described distribution in healthy muscle cells [49]. On the contrary, in muscle cells derived from Duchenne patients, dystrophin is located mainly in the nucleus. In the L6 ΔAQP4 cells, our results show a decrease in dystrophin levels at the plasma membrane with a primarily nuclear location, suggesting that the pair AQP4‐dystrophin at the plasma membrane is responsible not only for maintaining water homeostasis but also for preserving membrane integrity and regulating muscle signalling, functionality and differentiation tied to regeneration. With the observed decrease in muscle AQP4 expression with ageing, there may be a decrease in AQP4‐dystrophin complexes in the plasma membrane, which would impact membrane integrity and muscle function tied to functional decline with ageing.

Restoration of AQP4 expression in silenced muscle cells successfully reestablished key functional parameters, supporting its central role in muscle cell physiology. The complementation model demonstrated that AQP4 is essential for maintaining proper localization of dystrophin, sustaining Pax7 expression and enabling myotube formation. These experimental approaches were designed to validate the contribution of AQP4 to membrane integrity, signalling regulation and myogenic progression, independent of its classical role in water transport.

In conclusion, our findings identify AQP4 as a mechanistic driver of muscle ageing rather than a passive marker of degeneration. The consistent decline in AQP4 expression observed in aged muscle suggests that its loss precedes and potentially initiates key pathological changes associated with sarcopenia. Importantly, while dystrophin deficiency is linked to rare genetic disorders, the decline in AQP4 expression is a common feature of ageing muscle. Therefore, targeting AQP4 may represent a promising therapeutic strategy to counteract age‐related muscle atrophy, enhance muscle regenerative capacity and preserve muscle function in ageing populations.

## Funding

This work was supported by Abbott Laboratories S.A.

## Ethics Statement

All experimental procedures (approval code 16.02.2024.029) were carried out according to the European Convention for the Protection of Vertebrate Animals used for Experimental and other Scientific Purposes (Directive 2010/63/EU) and the ethical guidelines for animal experimentation provided by the Spanish National Research Council (RD 53/1 February 2013).

## Conflicts of Interest

This work was funded by Abbott Nutrition R&D. María D. Girón, Antonio Reinoso, Chiara Burgio, Nora Montero‐Martin, Guillermo Jiménez‐Jiménez and Josep M. Argilés declare that they have no conflicts of interest. Manuel Manzano, Suzette L. Pereira, Ricardo Rueda and Jose M. López‐Pedrosa are employees of the sponsor company Abbott Nutrition R&D.

## Supporting information


**Figure S1:** Ageing regulation of AQPs expression in two types of muscles. (A) Soleus (B) EDL muscle. Muscle lysates from adult and aged rats were used to measure the expression of AQPs proteins (*n* = 4). GAPDH was used as a loading control. Data are mean ± SEM *p < 0.05 vs. adult muscles.
**Figure S2:** PAX7 and CK levels in differentiated satellite cells*.* Satellite cells isolated from adult and aged rats were differentiated for 5 days in differentiation media and the expression of PAX7 and CK was analysed. GAPDH was used as a loading control. Results are mean ± SEM (*n* = 4). *p < 0.05 compared to undifferentiated adult or old satellite cells. #*p* < 0.05 compared to differentiated adult satellite cells.
**Figure S3:** Dexamethasone regulation of AQPs and differentiation markers in the gastronemius muscles. Rats were daily i.p. injected with 100 μg Dexamethasone/kg bw for 21 days. GAPDH was used as a loading control. Results are mean ± SEM (*n* = 8). *p < 0.05 compared to untreated rats.
**Figure S4:** Effects of AQP4 silencing on cell signalling in differentiated L6 and L6 ΔAQP4 cells. Akt, ERK and mTOR activation (*n* = 4). Data are mean ± SEM. *p < 0.05 vs. differentiated L6 cells.
**Figure S5:** Effects of AQP4 silencing on cell proliferation and differentiation in C2C12 and C2C12 ΔAQP4 cells. (A) Total AQP4 expression in normal (C2C12) and silenced (C2C12 ΔAQP4) cells. (B) Cell proliferation was measured for 72 h. Cell/field were counted every 24 h (*n* = 10). MTT was measured at 72 h (*n* = 8). (C) Myf5 and (D) Myf6 were measured by immunocytochemistry in C2C12 and C2C12 ΔAQP4 cells differentiated for 5 days (*n* = 25). Data are mean ± SEM. *p < 0.05 vs. C2C12 cells.
**Figure S6:** Propidium iodide uptake in L6 and L6 ΔAQP4 cells in the presence or absence of mannitol. Confocal images of L6 and L6 ΔAQP4 cells incubated in the absence or presence of 400 mM mannitol for 3 h and then incubated in the presence of 1 μg/mL propidium iodide for 15 min at room temperature. A quantification of propidium iodide staining is shown. Data (*n* = 10) are mean ± SEM. *p < 0.05 vs. L6 cells; #*p* < 0.05 vs. L6 ΔAQP4 cells.

**Table S1:** Antibodies used in the article.

## Data Availability

The paper and the supporting information present all the data needed to evaluate the conclusions.
