# Supplementary material for: Aquaporin‐4 Orchestrates Muscle Cell Differentiation and Satellite Cell Preservation: A Novel Target in Age‐Related Muscle Decline
Source: J Cachexia Sarcopenia Muscle. 2026 Oct 27;17(6):e70412. doi: 10.1002/jcsm.70412 (PMC13711248; doi:10.1002/jcsm.70412)
Supplement: Supplementary file 1 [file JCSM-17-e70412-s001.pdf]

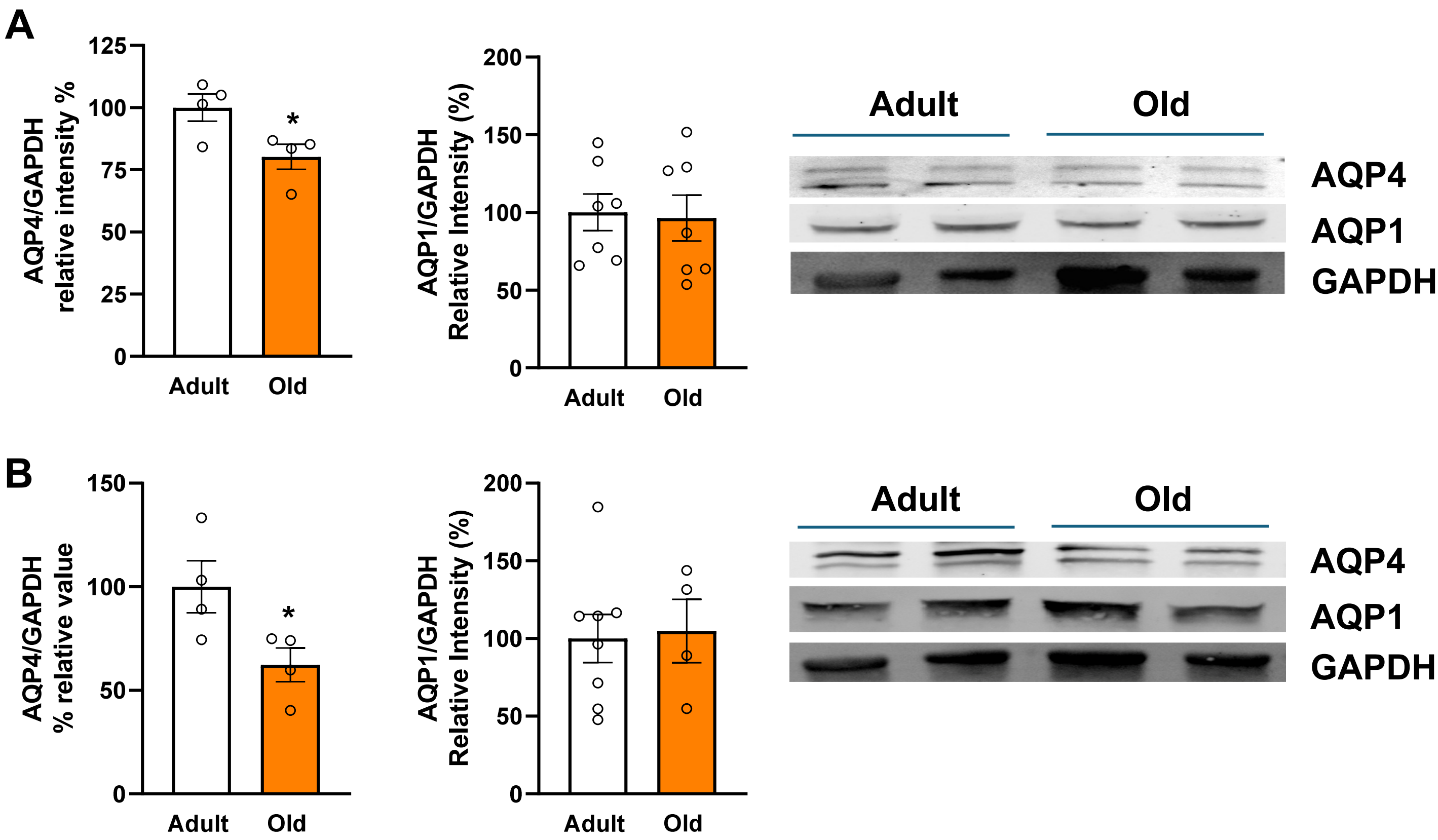

Fig. S1 | Aging regulation of AQPs expression in two types of muscles. (A) Soleus (B) EDL muscle. Muscle lysates from adult and aged rats were used to measure the expression of AQPs proteins (n=4). GAPDH was used as a loading control. Data are mean ± SEM. * $p<0.05$ vs adult muscles.

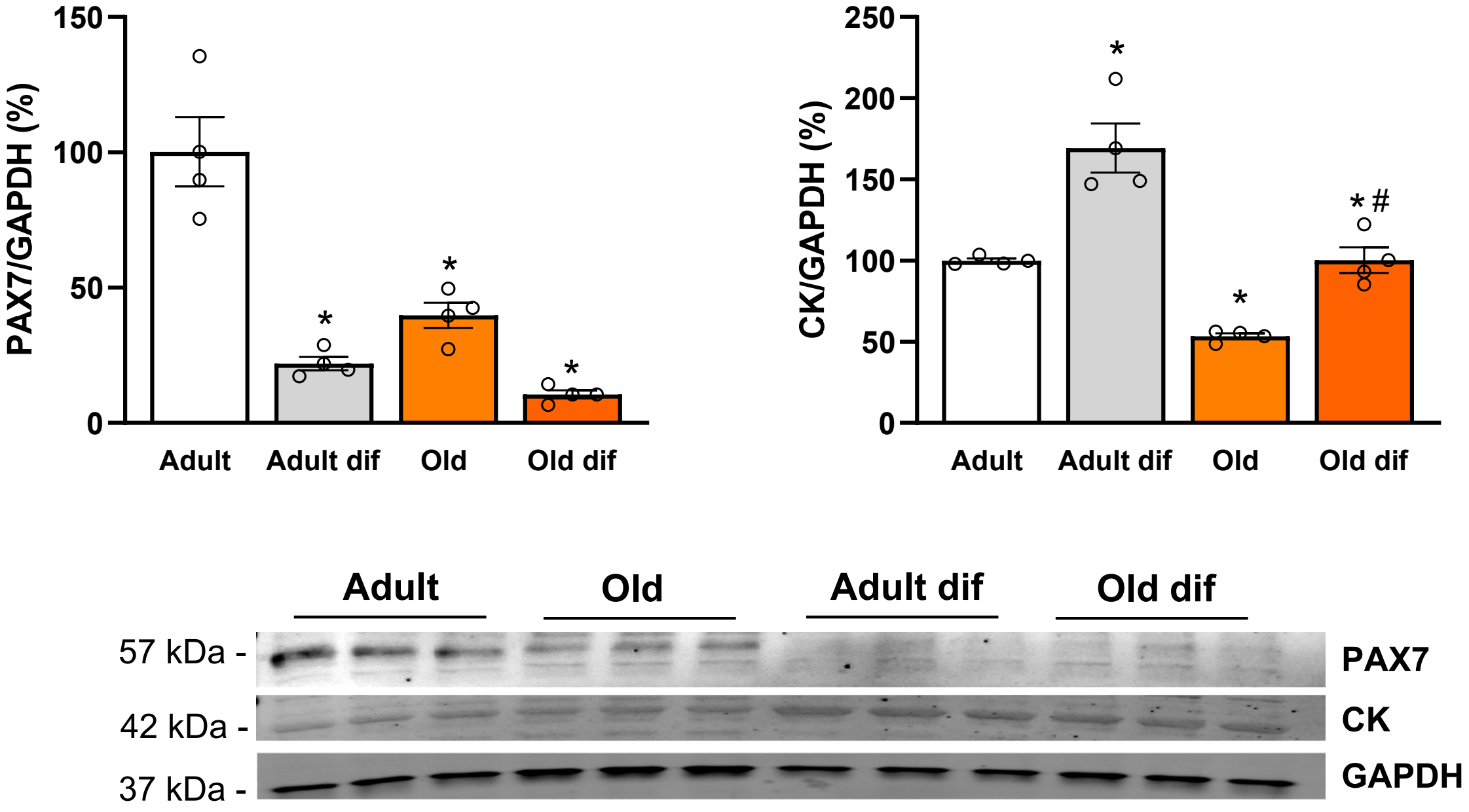

Fig. S2 | PAX7 and CK levels in differentiated satellite cells. Satellite cells isolated from adult and aged rats were differentiated for 5 days in differentiation media, and the expression of PAX7 and CK was analyzed. GAPDH was used as a loading control. Results are mean ± SEM (n=4). *$p<0.05$ compared to undifferentiated adult or old satellite cells. #$p<0.05$ compared to differentiated adult satellite cells.

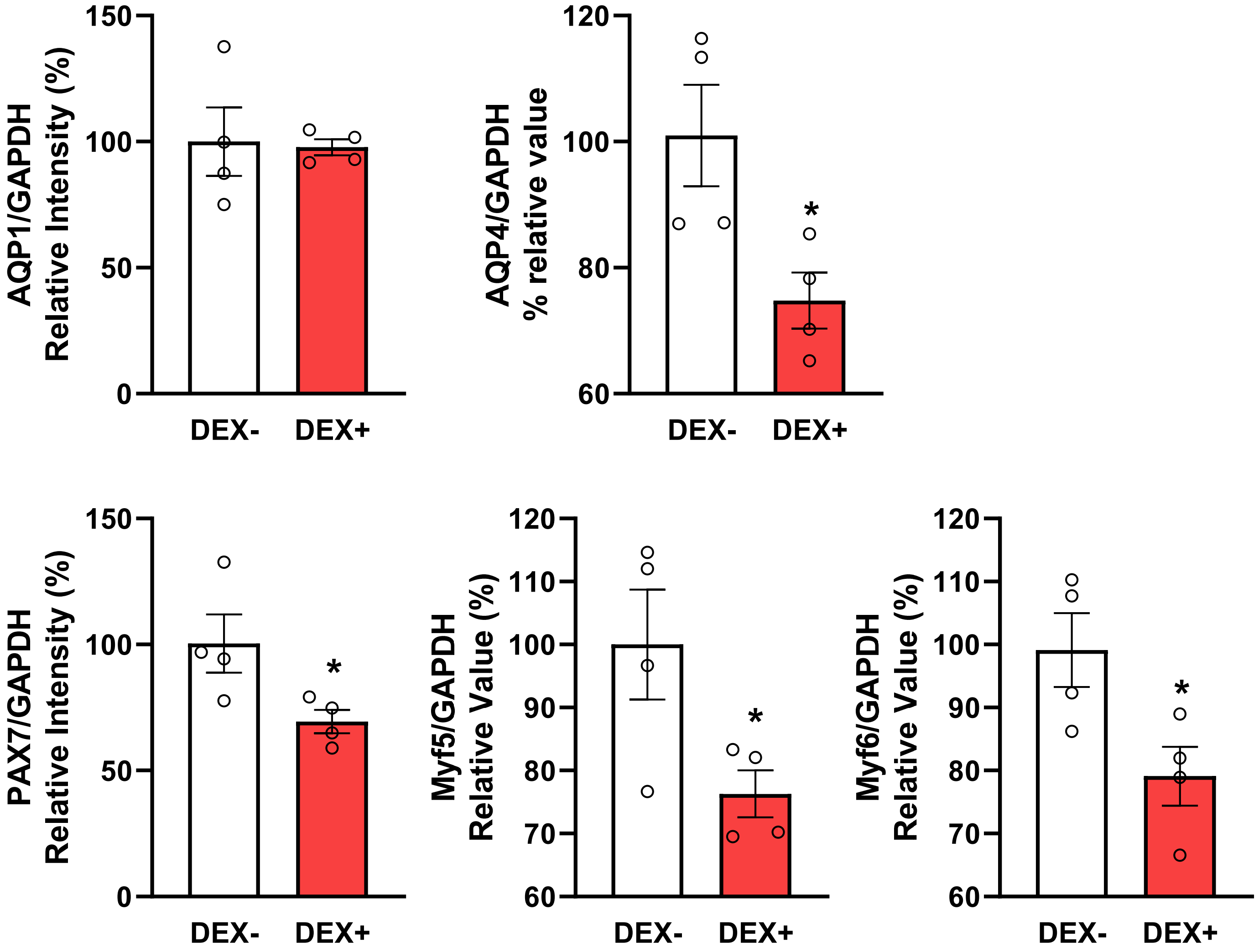

Fig. S3 | Dexamethasone regulation of AQPs and differentiation markers in the gastronemius muscles. Rats were daily i.p. injected with 100 µg Dexamethasone/kg bw for 21 days. GAPDH was used as a loading control. Results are mean ± SEM (n=8). *p<0.05 compared to untreated rats.

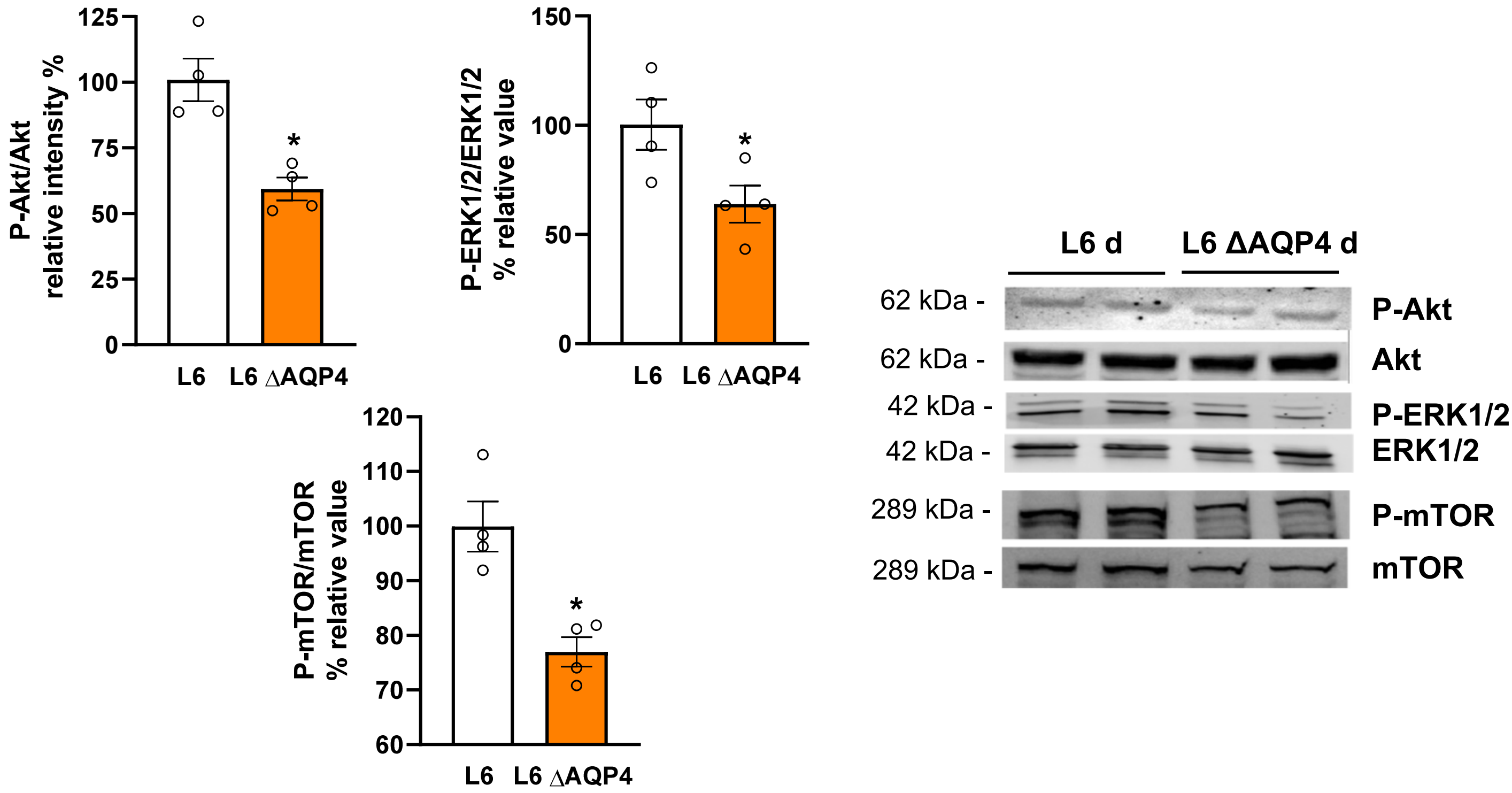

Fig. S4 | Effects of AQP4 silencing on cell signaling in differentiated L6 and L6 ΔAQP4 cells. Akt, ERK, and mTOR activation (n=4). Data are mean ± SEM. * $p<0.05$ vs differentiated L6 cells.

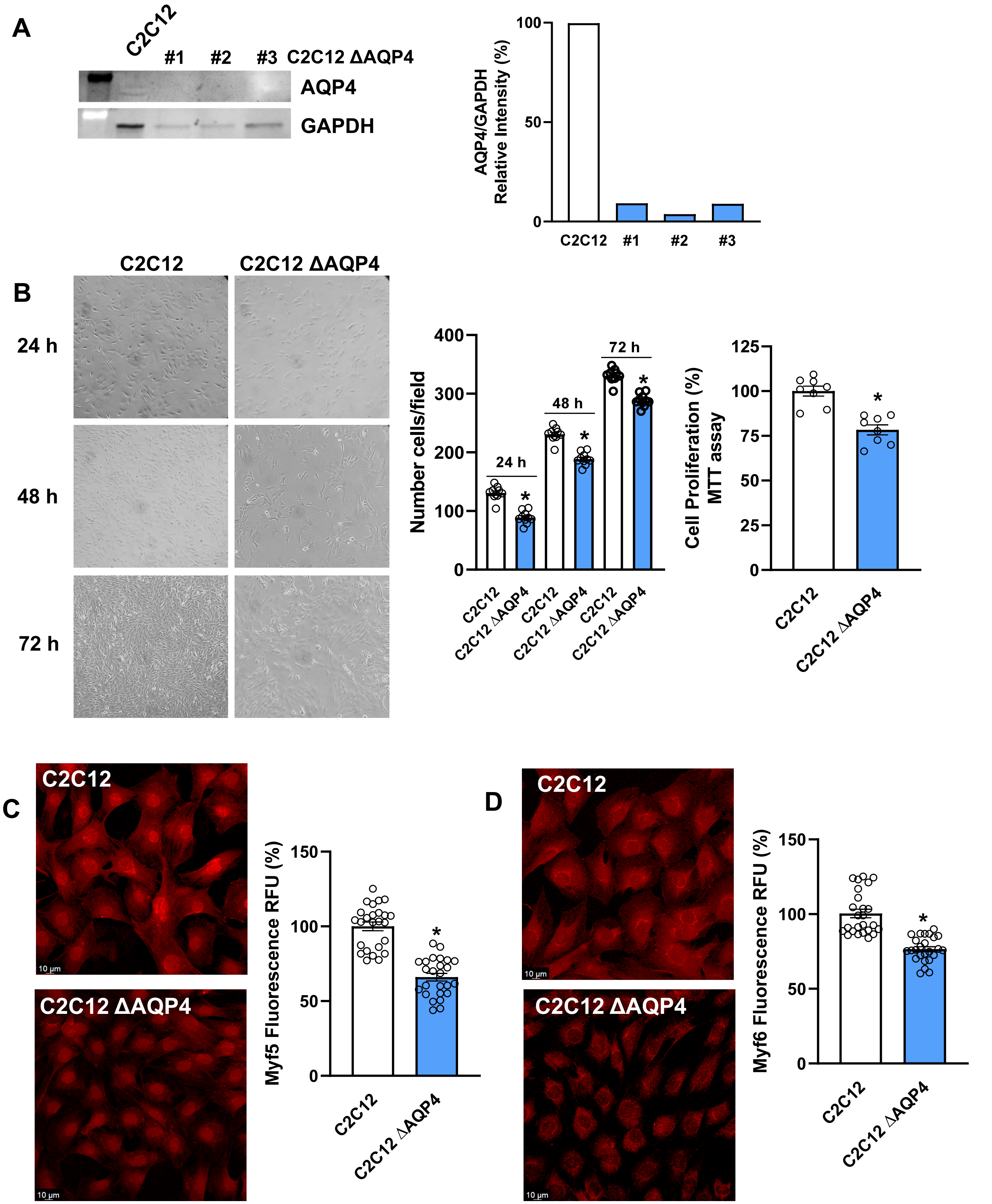

Fig. S5 | Effects of AQP4 silencing on cell proliferation and differentiation in C2C12 and C2C12 ΔAQP4 cells. (A) Total AQP4 expression in normal (C2C12) and silenced (C2C12 ΔAQP4) cells. (B) Cell proliferation was measured for 72 hours. Cell/field were counted every 24 hours (n=10). MTT was measured at 72 hours (n=8). (C) Myf5 and (D) Myf6 were measured by immunocytochemistry in C2C12 and C2C12 ΔAQP4 cells differentiated for 5 days. (n=25). Data are mean ± SEM. * $p<0.05$ vs C2C12 cells.

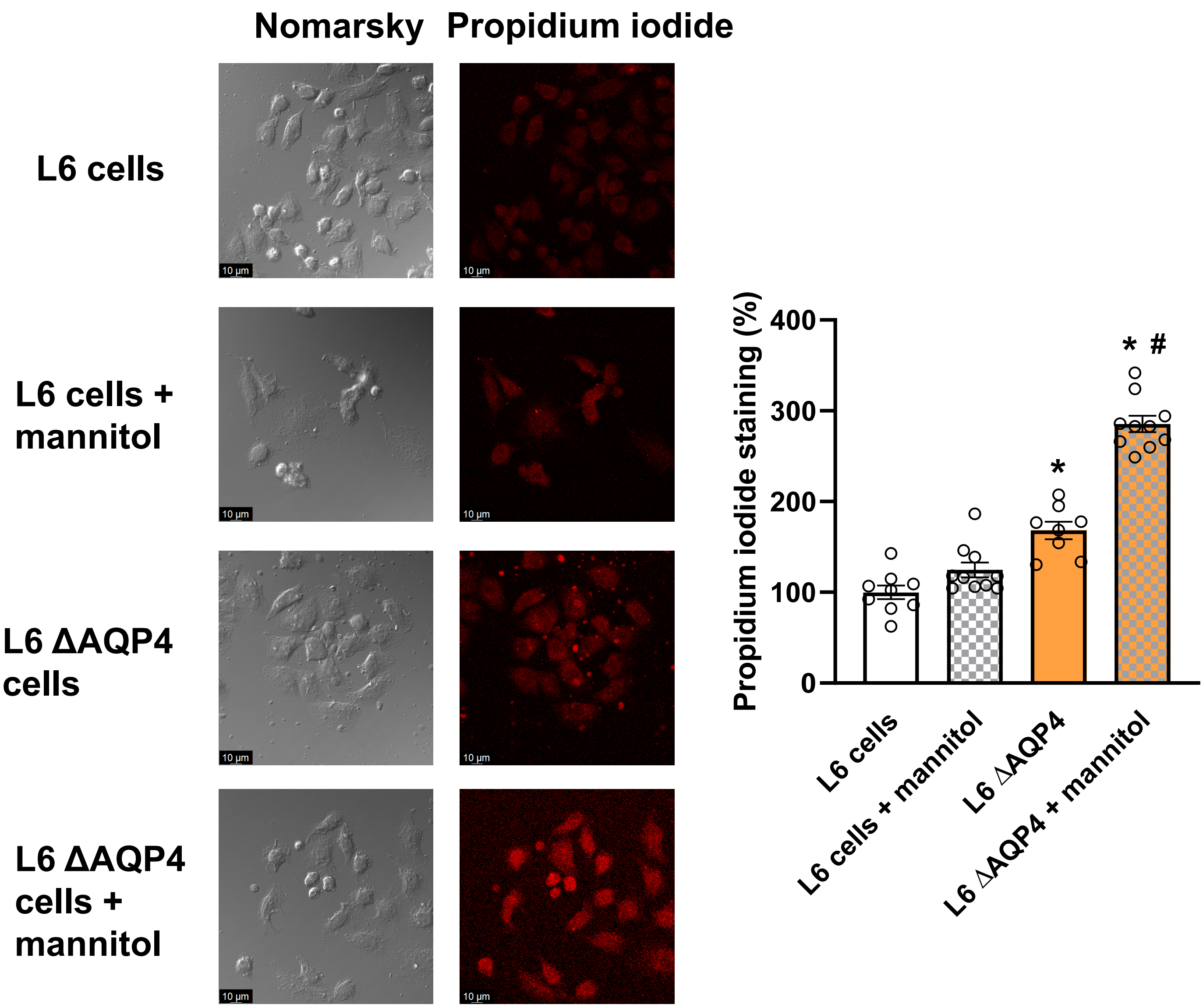

Fig. S6 | Propidium iodide uptake in L6 and L6 ΔAQP4 cells in the presence or absence of mannitol. Confocal images of L6 and L6 ΔAQP4 cells incubated in the absence or presence of 400 mM mannitol for 3 hours and then incubated in the presence of 1 µg/mL propidium iodide for 15 min at room temperature. A quantification of propidium iodide staining is shown. Data (n=10) are mean ± SEM. * $p<0.05$ vs L6 cells; # $p<0.05$ vs L6 ΔAQP4 cells.
