# Supplementary material for: Aquaporin‐4 Orchestrates Muscle Cell Differentiation and Satellite Cell Preservation: A Novel Target in Age‐Related Muscle Decline
Source: J Cachexia Sarcopenia Muscle. 2026 Oct 27;17(6):e70412. doi: 10.1002/jcsm.70412 (PMC13711248; doi:10.1002/jcsm.70412)
Supplement: Supplementary file 2 [file JCSM-17-e70412-s002.pdf]

Supplementary Table S1: Antibodies used in the article

Reagent or Resource	Source	Identifier
**Antibodies**		
Mouse Monoclonal anti-Akt1, Clone 5C10, 1:250 dilution	Santa Cruz Biotechnology	Cat# sc-81434, RRID:AB_1118808
Rabbit polyclonal Phospho-Akt (pSer473), 1:1000 dilution	Sigma-Aldrich	Cat# SAB4504331, RRID:AB_2893426
Mouse monoclonal anti-AQP1 (B-11), 1:500 dilution	Santa Cruz Biotechnology	Cat# sc-25287, RRID:AB_626694
Rabbit polyclonal anti-AQP4, 1:1000 dilution	Proteintech	Cat# 16473-1-AP, RRID:AB_2827426
Rabbit Polyclonal anti-Caveolin-1, 1:1000 dilution	Cell Signaling Technology	Cat# 3238, RRID:AB_2072166
Mouse monoclonal anti-Creatine kinase-M (G-9), 1:500 dilution	Santa Cruz Biotechnology	Cat# sc-365046, RRID:AB_10841424
Rabbit polyclonal anti-Dystrophin, 1:500 dilution	Thermo Fisher Scientific	Cat# PA5-16734, RRID:AB_10978300
Rabbit polyclonal p44/42 MAPK (Erk1/2) Antibody, 1:1000 dilution	Cell Signaling Technology	Cat# 9102, RRID:AB_330744
Mouse monoclonal anti-Phospho-p44/42 MAPK (Erk1/2) (Thr202/Tyr204) (E10), 1:500 dilution	Cell Signaling Technology	Cat# 9106, RRID:AB_331768
Mouse monoclonal anti-GAPDH (6C5), 1:5000 dilution	Santa Cruz Biotechnology	Cat# sc-32233, RRID:AB_627679
Rabbit polyclonal anti-SLC2A4, 1:1000 dilution	Sigma-Aldrich	Cat# SAB4300667, RRID:AB_11130232
Mouse monoclonal anti-MAFbx (F-9), 1:500 dilution	Santa Cruz Biotechnology	Cat# sc-166806, RRID:AB_2246982
Rabbit polyclonal anti-MEF2D, 1:500 dilution	Sigma-Aldrich	Cat# HPA007114, RRID:AB_1079353

Mouse monoclonal anti-mTOR (30), 1:500 dilution	Santa Cruz Biotechnology	Cat# sc-517464, RRID:AB_3186240
Rabbit polyclonal anti-Phospho-mTOR (Ser2448), 1:500 dilution	Thermo Fisher Scientific	Cat# 44-1125G, RRID:AB_2533580
Mouse monoclonal anti-MuRF1 (SW-53), 1:250 dilution	Santa Cruz Biotechnology	Cat# sc-134397, RRID:AB_2208860
Rabbit polyclonal anti-MYF5, 1:500 dilution	Thermo Fisher Scientific	Cat# PA5-115608, RRID:AB_2900243
Rabbit polyclonal anti-MYF6, 1:500 dilution	Thermo Fisher Scientific	Cat# PA5-51016, RRID:AB_2636464
Rabbit polyclonal anti-MYOD1, 1:500 dilution	Proteintech	Cat# 18943-1-AP, RRID:AB_10603467
Mouse Myosin 4 Monoclonal Antibody (MF20), 1:250 dilution	Invitrogen	Cat# 14-6503-82, RRID:AB_2572894
Rabbit polyclonal anti-p16INK4a, 1:250 dilution	Thermo Fisher Scientific	Cat# PA5-20379, RRID:AB_11157205
Rabbit polyclonal anti-p21, 1:500 dilution	Proteintech	Cat# 10355-1-AP, RRID:AB_2077682
Rabbit polyclonal anti-PAX7, 1:500 dilution	Sigma-Aldrich	Cat# SAB2103961, RRID:AB_10667238
Mouse monoclonal Anti-Troponin T, 1:500 dilution	Sigma-Aldrich	Cat# T6277, RRID:AB_261723
Goat Anti-Mouse IgG BioChemika Antibody, Atto 488 Conjugated	Sigma-Aldrich	Cat# 62197, RRID:AB_1137649
Goat Anti-Rabbit IgG BioChemika Antibody, Atto 594 Conjugated	Sigma-Aldrich	Cat# 77671, RRID:AB_1137663
